# 2177. Characterization of community-acquired methicillin-resistant *Staphylococcus aureus* isolated from patients with skin and soft tissue infections in selected health facilities, in Kenya

**DOI:** 10.1093/ofid/ofad500.1799

**Published:** 2023-11-27

**Authors:** John N Njenga

**Affiliations:** Jomo kenyatta university of agriculture and technology, Nairobi, Nairobi Area, Kenya

## Abstract

**Background:**

Colonization of the skin, mucus membrane, and nose of humans by *Staphylococcus aureus (S. aureus)* is more common than infection. The lack of proper microbiological surveillance systems in Africa may underestimate the burden of Community-acquired methicillin-resistant *Staphylococcus aureus* (CA-MRSA), while little is known about its incidence. In Kenya, there is a paucity of data on prevalence and circulating CA-MRSA strains.

**Methods:**

Cross-sectional study design was employed with a sample size of 228 wound swabs from community-acquired skin and soft tissue infections, collected from 5 AMR surveillance sites in Kenya. *S. aureus* isolation, identification, and antimicrobial susceptibility tests were carried out according to standard bacteriology methods. Molecular characterization for MRSA isolates was performed by whole genome sequencing.

**Results:**

Descriptive and inferential statistics were computed using the R program. Total *S. aureus* isolated was 29.38 % while MRSA isolation varied within study sites, with a CA-MRSA prevalence of 1.8 %. Phenotypic resistance observed did not match genotypic resistance for some isolates. Resistance mostly reported in nosocomial infections and resistance to reserved antibiotics was observed, which raises concern. Mutations within resistance genes, Inducible-Macrolide-Lincosamide-Streptogramin b resistance (MLSBi), 23S ribosome resistance and also erythromycin resistance by efflux pump was observed. Staphylococcal chromosomal cassette mec (SCC*mec*) type IV was the most prevalent while the sequence types (STs) obtained were ST 30, 8 and the novel STs 7460 and 7635. Only one isolate had intact *mecA* and its regulatory genes, but still conferred methicillin resistance. IS 1272 was detected in *mecA* regulatory genes.

**Conclusion:**

The resistance patterns observed in this study further highlight the burden of antimicrobial resistance in community isolates of *S. aureus*. There is need for more research on CA-MRSA within Antimicrobial resistance (AMR) surveillance in Kenya in order to better understand the molecular epidemiology as well as the drivers of resistance

**Disclosures:**

**All Authors**: No reported disclosures

